# Hsa_circ_0046264 up-regulated *BRCA2* to suppress lung cancer through targeting hsa-miR-1245

**DOI:** 10.1186/s12931-018-0819-7

**Published:** 2018-06-11

**Authors:** Liu Yang, Jun Wang, Yaodong Fan, Kun Yu, Baowei Jiao, Xiaosan Su

**Affiliations:** 1 Biomedical Research Center, the Affiliated Calmette Hospital of Kunming Medical University (the First Hospital of Kunming), No. 504 Qingnian Road, Kunming, 650011 Yunnan China; 2 Department of Anesthesiology, the First Affiliated Hospital of Kunming Medical University, Kunming, 650032 Yunnan China; 3 Department of Neurosurgery, the Third Affiliated Hospital of Kunming Medical University (Yunnan Cancer Hospital), Kunming, 650118 Yunnan China; 4 Department of Colorectal Cancer, the Third Affiliated Hospital of Kunming Medical University (Yunnan Cancer Hospital), Kunming, 650118 Yunnan China; 5 State Key Laboratory of Genetic Resources and Evolution, Kunming Institute of Zoology, Chinese Academy of Sciences, Kunming, 650223 Yunnan China

**Keywords:** hsa_circ_0046264, Hsa-miR-1245, *BRCA2*, Lung cancer

## Abstract

**Objective:**

Lung cancer had been leading mounts of deaths worldwide. Advances in genes microarray had helped human further understand genes and identify novel circular RNAs. This study aimed at investigating the biological functions and molecular mechanisms of hsa_circ_0046264 in lung cancer which may be helpful in lung cancer early diagnosis and clinical treatment.

**Methods:**

Gene microarray data screened the differential gene of hsa_circ_0046264 and its downstream genes were found by bioinformatics analysis and verified by luciferase reporter assay. QRT-PCR and Western blot was used to detect the RNA and protein levels respectively. RNase R digestion confirmed the existences of circular RNA. Cell viability, invasion and apoptosis were determined by MTT assay, flow cytometry and DNA damage assay. Tumor formation in nude mice and immunohistochemistry proved the functions of hsa_circ_0046264 in vivo.

**Results:**

Hsa_circ_0046264 and *BRCA2* were down-regulated in lung cancer tissues while miR-1245 was up-regulated. Hsa_circ_0046264 induced apoptosis but inhibited proliferation and invasion of lung cancer cells through targeting miR-1245 to up-regulate BRCA2. Hsa_circ_0046264 inhibited the tumor growth in vivo.

**Conclusion:**

Hsa_circ_0046264 was a tumor suppressor in lung cancer. Overexpression of hsa_circ_0046264 could up-regulate *BRCA2* expression through down-regulating of miR-1245.

**Electronic supplementary material:**

The online version of this article (10.1186/s12931-018-0819-7) contains supplementary material, which is available to authorized users.

## Introduction

Lung cancer mortality ranks the highest in cancer-related death, and the five-year survival rate is only about 20% [1]. The development of target small molecule inhibitors provides an alternative treatment strategy to chemotherapy for lung cancer patients, with improved survival rate and life quality [2, 3]. The exploration of the molecular mechanisms in lung cancer is very important for developing new strategies of diagnosis and treatment to this disease [4]. Novel therapeutic targets together with prognostic biomarkers for lung carcinogenesis have been discovered due to the development of molecular biology techniques [5]. Therefore, detailed understanding of the molecular mechanisms and searching for innovative targets are essential for the improvement of lung cancer treatment.

Circular RNAs (circRNAs) are featured with enormous abundance, evolutional conservation and relative stablity in cytoplasm that confer numerous potential functions, such as acting as competing endogenous RNAs (ceRNAs) for encoding RNAs, sponging microRNAs (miRNAs) to regulate miRNAs’ downstream genes [6, 7]. Hansen et al. identified ciRS-7, a circular RNA, which acts as a designated miR-7 inhibitor/sponge, has conceptually changed the mechanistic understanding of miRNA networks in cancers [8]. With binding capacities to miRNAs, some circRNAs were demonstrated to have great influence on lung cancer progression. For instance, significant down-regulation of circRNA *ITCH* was discovered in lung cancer and up-regulating its expression could markedly elevate its parental cancer-suppressive gene *ITCH* through sponging oncogenic miR-7 and miR-214 [9]. In non-small cell lung cancer, hsa_circ_0043256 could be up-regulated by cinnamaldehyde and functioned as a miR-1252 sponge to positively affect *ITCH* expression [10]. Hsa_circ_0013958 was up-regulated in lung adenocarcinoma and identified as a sponge of miR-134, thus up-regulating oncogenic cyclin D1 [11]. Since studies on circRNAs are not sufficient, in-depth researches about their functions in lung cancer are still needed.

As small (up to 21 nucleotides) noncoding RNAs, miRNAs can regulate their downstream genes through binding to the 3’UTRs of the target genes [12]. MiR-1245 was considered as an oncogene because it was demonstrated as a potent negative regulator of the tumor suppressor protein BRCA2 [13]. MiR-1245 attenuated the expression of *NKG2D*, an activating receptor involved in tumor immunosurveillance, in natural killer cells [14]. It directly targeted *BRCA2* and suppressed its translation in breast cancer [15]. As a tumor suppressor gene, *BRCA2* mutation was reported to be associated with breast cancer and ovarian cancer development [16, 17]. Its expression was frequently down-regulated in lung adenocarcinomas [18]. Therefore, miR-1245 and its target gene *BRCA2* might affect the progression of lung cancers.

In the present study, we evaluated the expression profiles of circRNAs in lung cancer tissues and found the significant downregulation of hsa_circ_0046264. The biological functions of hsa_circ_0046264 and its regulatory relationships with miR-1245 and *BRCA2* were investigated. The results indicated the suppressive effect of hsa_circ_0046264 in lung cancer and provided novel insights into the crucial role of circRNA.

## Materials and methods

### Microarray processing

The expression profiles of circRNAs in 10 tissue samples (5 lung cancer tissues and 5 non-tumor adjacent tissues) were analyzed by microarray GSE101586 from GPL19978 platform. Five female lung patients with no smoking history were selected to profile circular RNA expression with microarrays. CircRNAs differentially expressed between tumor and normal tissues were identified via fold change filtering and *P* value. The screening condition was fold change > 2 and *P* < 0.05. Hierarchical clustering was performed to show the distinguishable circRNA expression pattern among samples.

### Participants and tissue samples

In total, 99 lung cancer tissue specimens, containing 23-paired specimens, were obtained from Affiliated Calmette Hospital of Kunming Medical University (Yunnan, China). The pathological information refered in this paper was provided in Table 1. Among them, 23 pared specimens included 5 Stage I tissues without lymphatic metastasis, 4 Stage II tissues, 6 Stage III tissues and 8 Stage IV tissues. The corresponding adjacent nontumorous tissues were taken 5 cm from the edge of the cancer and contained no obvious tumor cells. All specimens were confirmed as lung cancer by 3 independent pathologists. No patient received chemotherapy or other treatments for lung cancer before surgery. Tissues were acquired and immediately frozen in liquid nitrogen and stored at − 80 °C until use. Follow-up information of all the patients was collected through office visits or telephone interviews either with the patients or with a relative. The time of follow-up was calculated until death or last contact in October 2017. This study was approved by the Ethical Review Board for Research in the Affiliated Calmette Hospital of Kunming Medical University.

**Table 1:** Correlation between expression of circRNA_0046264 and clinic pathological features in lung cancer patients

Parameters	Group	Total	circRNA_0046264		*P*
			High	Low	
Age	≤ 60	46	24	22	0.528
	>60	53	31	22	
Gender	Female	23	12	11	0.710
	Male	76	43	33	
Tumor diameter	≤5 cm	41	25	16	0.362
	>5 cm	58	30	28	
Staging	I	56	38	18	0.0003^b^
	II	10	7	3	
	III	12	4	8	
	IV	21	6	15	
Tumor differentiation	well	7	5	2	0.111
	moderate	73	36	37	
	poor	19	14	5	
Pathological type	Squamous cell carcinoma	36	17	19	0.207
	Adenocarcinoma	63	38	25	
Lymphatic metastasis	Yes	23	7	16	0.006^b^
	No	76	48	28	

^a^Low and High expression group were divided according to the median ratio of relative circRNA_0046264 expression

^b^*P* value was determined by chi-square analysis. *P*<0.05 was statistically significant

### Cell lines and cell culture

Cell lines included human highly metastasis lung cancer cell line 95-D, common lung cancer cell line A549, normal human lung cell line HLF-a and normal human embryonic lung fibroblast line MRC-5. 95-D and A549 were provided by the Cell Bank of Type Culture Collection of the Chinese Academy of Sciences (Shanghai, China). HLF-a and MRC-5 were purchased from BeNa Culture Collection (Beijing, China). 95-D cells were cultured in RPMI-1640 (GIBCO, Grand Island, NY, USA) and 10% FBS (GIBCO), while MRC-5, HLF-a and A549 cells were cultured in high-glucose DMEM (Thermo Fisher Scientific, Waltham, MA, USA) and 10% FBS. Cells were incubated at 37 °C in a humidified atmosphere containing 5% CO_2_.

### RNase R digestion

Total RNA (5 μg) was incubated for 15 min at 37 °C with 3 U/ug of RNase R (Epicentre Biotechnologies, Madison, WI, USA). The RNase R digestion reaction was performed twice following previously published procedures [19].

### QRT-PCR

200 ng total RNA extracted by TRIzol® reagent (Invitrogen, Carlsbad, CA, USA) and quantified by NanoDrop 2000 (Thermo Fisher Scientific) was reversely transcribed by ReverTra Ace qPCR RT Kit (Toyobo, Japan). Quantitative RT-PCR was performed on THUNDERBIRD SYBR® qPCR Mix (Toyobo). Briefly, the reaction was initiated in a 96-well optical plate at 94 °C. After 2 min, 30 cycles were conducted under the conditions of 94 °C for 30 s, 56 °C for 30 s and 72 °C for 1 min. Finally, the reaction ended at 72 °C for 10 min. The expression level was calculated by 2^− △  △ *CT*^ method with GADPH and U6 as internal controls. The primers used for real-time PCR were shown in Table 2.

**Table 2:** Primers of qRT-PCR

GENE	PRIMER	SEQUENCES
hsa_circ_0046264	Forward primer	CGACAAAGATGGGGTTGTCC
	Reverse primer	CCAACCTGATCTCGGAACCT
	Product length	124 bp
BRCA2	Forward primer	CAGGTAGACAGCAGCAAGCA
	Reverse primer	AAGCCCCTAAACCCCACTTC
	Product length	134 bp
GAPDH	Forward primer	TCGGAGTCAACGGATTTGGT
	Reverse primer	TTCCCGTTCTCAGCCTTGAC
	Product length	181 bp
miR-1245	Forward primer	GGCCTGAAGTGATCTAAAGG
	Reverse primer	GTATCCAGTGCGAATACCTC
	Product length	71 bp
U6	Forward primer	CTCGCTTCGGCAGCACA
	Reverse primer	AACGCTTCACGAATTTGCGT
	Product length	94 bp

### Cell transfection

Plasmid vector pLO-ciR used for hsa_circ_0046264 overexpression, miR-1245 mimics, miR-1245 inhibitor and negative control miR-con were purchased from GENESEED (Guangzhou, China). SiRNAs for knockdown hsa_circ_0046264 was purchased from Geneseed Biotech (Guangzhou, China). The transfection was conducted using Lipofectamine™ 2000 (Invitrogen) in 6-well plates with 500 ng–1000 ng plasmid. The concentration of miR-1245 mimics or miR-1245 inhibitor was 30 nM–50 nM. The sequence of hsa_circ_0046264 was obtained from circbase.

### Cell proliferation

Lung cancer cells transfected with hsa_circ_0046264 were plated into 96-well plates (1 × 10^3^ cells / well). At 24, 48 and 72 h post-transfection, 10 μl MTT with the concentration of 5 mg/ml and pH of 7.4 were added into cells and cultured for 4 h at 37 °C, followed by removal of the culture medium and the addition of 100 μl dimethyl sulfoxide (DMSO, Sigma-Aldrich, St. Louis, MO, USA). The absorbance at 450 nm was measured with a microplate reader (Molecular Devices, Sunnyvale, CA, USA).

### Cell apoptosis

Lung cancer cells transfected with hsa_circ_0046264 were collected and treated with Annexin V-FITC/PI Cell Apoptosis Detection Kit (YEASEN, Shanghai, China) in accordance with the manufacturer’s instructions. Cell apoptosis was then detected by flow cytometry (FCM) FACS Calibur (BD Biosciences, CA, USA) and analyzed by software FACS Diva.

### Cell invasion

Invasive capacity was detected using Transwell cell culture inserts (8- mm pores; Corning, NY, USA) in 24-well plates. After transfection, cells were resuspended in serum-free medium and added to chambers coated with Matrigel (BD Biosciences). The bottom well contained growth medium with 20% FBS. After 48 h incubation, invaded cells were fixed with methanol and stained with hematoxylin. The invaded cells were counted under a microscope.

### Dual luciferase reporter assay

The reporter vector pmirGLO (Crisprbio, Beijing, China) was inserted with hsa_circ_0046264 or 3’UTR sequence of *BRCA2* (the sequence was obtained from UTRdb) to construct recombinant pmirGLO. HEK293 cells (Thermo Fisher Scientific) transfected with miR-1245 mimics or miR-con were seeded in 24-well plates and co-transfected with empty pmirGLO or recombinant pmirGLO for 48 h by using Lipofectamine™ 2000 (Invitrogen). Recombinant vectors containing wild types of hsa_circ_0046264 were named as WT. MUT1s were deletion mutations based on WT while MUT2s were point mutations. After transfection, cells were lysed by 1× Passive Lysis Buffer (80 μl for each well) and separated by centrifugation. Dual-Luciferase Reporter System (Promega, Madison, WI, USA) was applied to measure their Firefly and Renilla luciferase activities.

### DNA damage assay

After transfected by hsa_circ_0046264 for 48 h, lung cancer cells were digested by pancreatin and fixed on glass slides by polylysine. The cells were incubated with 25 μM Etoposide for 5 h (Treated) or solvent-only for control (Non-treated). Cells were fixed with 4% formaldehyde (10 min) and then blocked in 1% BSA/10% normal goat serum/0.3 M glycine in 0.1% PBS-Tween for 1 h. The cells were then incubated with ab206900 staining Histone H2A.X (phospho S139) at 1/100 dilution (shown in red) overnight at 4 °C. Nuclear DNA was labelled in blue with DAPI. The fluorescence intensity was observed and photographed using a fluorescence microscope (Olympus, CX23, Japan).

### Xenograft nude mouse model

Male BALB/c nude mice (*n* = 10, 5–8 weeks old, 18–20 g) were obtained from Experimental Animal Research Center of the Affiliated Calmette Hospital of Kunming Medical University. Empty or recombinant pLO-ciR was transfected into 95-D lung cancer cells, which was then injected into the posterior axilla of nude mice (1 × 10^7^ cells / mL, 0.2 mL for each mouse). The mice were randomly divided into two groups: control group injected with empty pLO-ciR (*n* = 5) and over-circ group injected with recombinant pLO-ciR (n = 5). After 2 weeks, tumors were measured every week and calculated by the formula V = ab^2^/2 (a represents tumor length and b represents tumor width). After 7 weeks, mice were killed and tumors were collected and weighted. Total RNA of tumor tissues was extracted by TRIzol reagent for qRT-PCR. The tumor tissues were embedded by paraffin for immunohistochemistry. All procedures were performed following the ethical standards and under the protocol approved by the Affiliated Calmette Hospital of Kunming Medical University.

### Immunohistochemistry

Tumor tissue sections from nude mice were deparaffinized and rehydrated with xylene and ethanol solutions, respectively. They were soaked in citrate buffer (pH = 6.0) and heated by microwave at 95 °C for 3 min twice to retrieve antigen and incubated in 3% H_2_O_2_ for 10 min after cooling down. Then sections were incubated with nonimmune goat serum for 15 min, followed by primary and secondary antibodies incubation at 4 °C for 12 h and 30–40 min respectively. The primary antibody was anti-*BRCA2* (ab27976, Abcam, Cambridge, MA, USA) diluted to 1:100 and the secondary one was goat anti-rabbit IgG-HRP (1:1000, ab6721, Abcam). After washing by PBS, each section was added with 1 drop newly prepared diaminobenzidine for coloration (20 min) and hematoxylin for counterstain (30 s). Finally, after being dehydrated with ethanol, sections were transparentized by xylene, sealed with resinene and air dried for observation.

### Statistical analysis

Software GraphPad Prism 6.0 was used for data analysis. Values were expressed by mean ± standard deviation. Student’s *t* test and One-Way ANOVA were used to evaluate significance between two groups and among multiple groups, respectively. All experiments were independently repeated three times. *P* < 0.05 meant statistically significance.

## Results

### Hsa_circ_0046264 was down-regulated in lung cancer

**Fig. 1:**
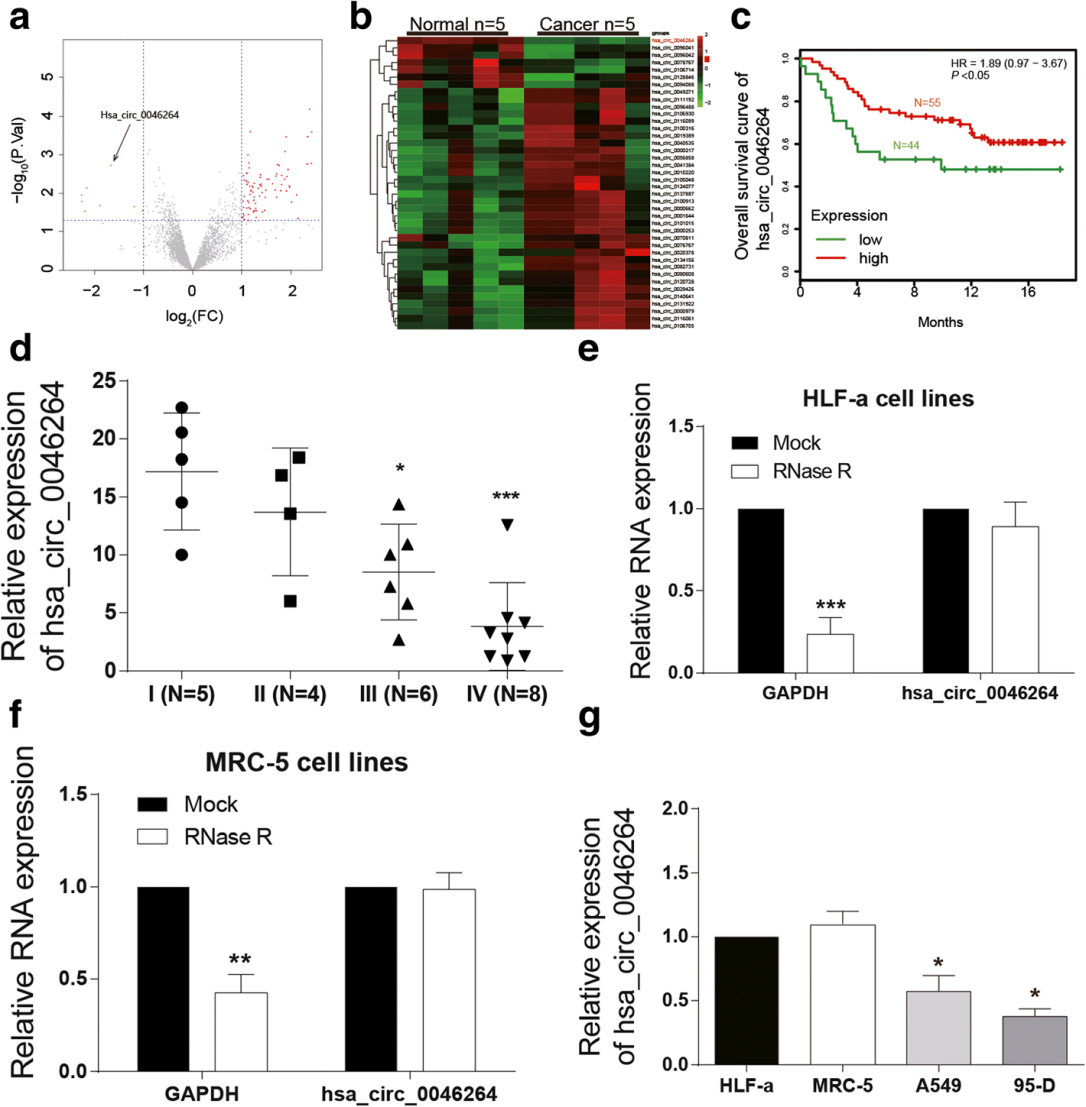
Hsa_circ_0046264 was down-regulated in lung cancer tissues. **a** Differentially expressed circRNAs in lung cancer tissues were presented in a volcano plot. The screening condition was fold change > 2 and *P* < 0.05. **b** The heat map reflected 50 significantly differentially expressed circRNAs in lung cancer tissues. Hsa_circ_0046264 was lowly expressed in cancer tissues. **c** The overall survival (OS) of lung cancer patients with high or low circ_0046264 level. A low level predicted a worse prognosis outcome. **d** The expression level of hsa_circ_0046264 decreased with the deterioration of lung cancer (from stage I to stage IV). ^*^P < 0.05, ^***^P < 0.001, compared with I (N = 5) group with lung cancer tissues in Stage I. **e**-**f** RNase R can digest liner RNA GAPDH but not circRNA hsa_circ_0046264 as the relative expression of GADPH in lung cells decreased after RNase R digestion while that of hsa_circ_0046264 had no significant difference. ^**^P < 0.01, ^***^P < 0.001, compared with MOCK group. **g** The relative expression of hsa_circ_0046264 was lower in lung cancer cells A549 and 59-D than that in normal lung cells. ^*^P < 0.05 compared with HLF-a cells

Bioinformatic analysis screened out differential expressed circRNAs in lung cancer tissues (Fig. 1a) while 50 among them with the most significant differential expressions were displayed in Fig. 1b. Hsa_circ_0046264 was significantly down-regulated in tumor tissues (Fig. 1b). Low hsa_circ_0046264 level predicted a better survival outcome (Fig. 1c). The expression levels of hsa_circ_0046264 in lung cancer tissues with different tumor stages also proved its low expression, and further indicated that hsa_circ_0046264 expression decreased with the deterioration of lung cancer (Fig. 1d).

To further investigate the regulatory mechanism, 19 potential target miRNAs of hsa_circ_0046264 were screened out by the website circinteractome (Table 3). Furthermore, three binding sites of AGO2 protein (the protein that widely expresses in organisms and serves as a crucial composition of RNA induced silencing complex) on hsa_circ_0046264 were found (Table 4). The target gene of miR-1245 was determined as *BRCA2* through the database miRTarBass. In both HLF-a and MRC-5 cell lines, after RNase digestion, the content of GAPDH significantly decreased, whereas the content of has_circ_0046264 did no significantly differ from that before RNase digestion (Fig. 1e and f). Compared with normal human lung cell lines MRC-5 and HLF-a, the expression of circ_0046264 was much lower in human lung cancer cell lines A549 and 95-D (Fig. 1g).

**Table 3:** Target miRNAs of hsa_circ_0046264 with the web of https://circinteractome.nia.nih.gov/

CircRNA Mirbase ID	CircRNA (Top) - miRNA (Bottom) pairing	Site Type	CircRNA Start	CircRNA End	context+ score percentile
hsa_circ_0046264 (5′ ... 3′)	CUGCCUGACGGCGCAGCUGCAGA	8mer-1a	288	295	95
	|||||||				
hsa-miR-1184 (3′ ... 5′)	CCUUCGGUAGUUCAGCGACGUCC				
hsa_circ_0046264 (5′ ... 3′)	CCACCAUCAAGUUCUUCAGGAAU	7mer-1a	169	175	81
	||||||				
hsa-miR-1200 (3′ ... 5′)	CUCCGAGUCUUACCGAGUCCUC				
hsa_circ_0046264 (5′ ... 3′)	UGCCUGACGGCGCAGCUGCAGAG	7mer-1a	289	295	64
	||||||				
hsa-miR-1205 (3′ ... 5′)	GAGUUUCGUUUGGGACGUCU				
hsa_circ_0046264 (5′ ... 3′)	UUUGGGAUCACUUCCAACAGUGA	8mer-1a	420	427	98
	|||||||				
hsa-miR-1208 (3′ ... 5′)	AGGCGGACAGACUUGUCACU				
hsa_circ_0046264 (5′ ... 3′)	UGACAUACCAUUUGGGAUCACUU	7mer-m8	410	416	93
	|||||||				
hsa-miR-1245 (3′ ... 5′)	UACAUCCGGAAAUCUAGUGAA				
hsa_circ_0046264 (5′ ... 3′)	AAAGAUGGGGUUGUCCUCUUUAA	7mer-1a	468	474	86
	||||||				
hsa-miR-1276 (3′ ... 5′)	ACAGAGGUGUCCCGAGAAAU				
hsa_circ_0046264 (5′ ... 3′)	AUCAAGUUCUUCAGGAAUGGAGA	8mer-1a	174	181	94
	|||||||				
hsa-miR-136 (3′ ... 5′)	AGGUAGUAGUUUUGUUUACCUCA				
hsa_circ_0046264 (5′ ... 3′)	UCUGGCCCCUGAGUAUGCCAAAG	7mer-1a	44	50	72
	||||||				
hsa-miR-182 (3′ ... 5′)	UCACACUCAAGAUGGUAACGGUUU				
hsa_circ_0046264 (5′ ... 3′)	GUUCUUCAGGAAUGGAGACACGG	7mer-m8	179	185	76
	|||||||				
hsa-miR-187 (3′ ... 5′)	GGCCGACGUUGUGUUCUGUGCU				
hsa_circ_0046264 (5′ ... 3′)	AGACACGGCUUCCCCCAAGGAAU	7mer-1a	194	200	73
	||||||				
hsa-miR-502-5p (3′ ... 5′)	AUCGUGGGUCUAUCGUUCCUA				
hsa_circ_0046264 (5′ ... 3′)	UGCAAGGCUCUGGCCCCUGAGUA	8mer-1a	36	43	98
	|||||||				
hsa-miR-510 (3′ ... 5′)	CACUAACGGUGAGAGGACUCAU				
hsa_circ_0046264 (5′ ... 3′)	GCAAGGCUCUGGCCCCUGAGUAU	7mer-1a	37	43	79
	||||||				
hsa-miR-512-5p (3′ ... 5′)	CUUUCACGGGAGUUCCGACUCAC				
hsa_circ_0046264 (5′ ... 3′)	CAAAGAUGGGGUUGUCCUCUUUA	8mer-1a	467	474	99
	||||| |||||||				
hsa-miR-583 (3′ ... 5′)	CAUUACCCUGGAAGGAGAAAC				
hsa_circ_0046264 (5′ ... 3′)	CCAACAGUGACGUGUUCUCCAAA	7mer-1a	433	439	89
	||||||				
hsa-miR-1270 (3′ ... 5′)	UGUGUCGAGAAGGUAUAGAGGUC				
hsa_circ_0046264 (5′ ... 3′)	CCAACAGUGACGUGUUCUCCAAA	7mer-1a	433	439	86
	||||||				
hsa-miR-620 (3′ ... 5′)	UAAAGAUAUAGAUAGAGGUA				
hsa_circ_0046264 (5′ ... 3′)	GAAUGGAGACACGGCUUCCCCCA	8mer-1a	188	195	97
	|||||||				
hsa-miR-625 (3′ ... 5′)	CCUGAUAUCUUGAAAGGGGGA				
hsa_circ_0046264 (5′ ... 3′)	CAGGAAUGGAGACACGGCUUCCC	7mer-m8	185	191	82
	|||||||				
hsa-miR-671-5p (3′ ... 5′)	GAGGUCGGGGAGGUCCCGAAGGA				
hsa_circ_0046264 (5′ ... 3′)	AAGCGCACGGGCCCGGCUGCCAC	7mer-1a	261	267	63
	||||||				
hsa-miR-885-3p (3′ ... 5′)	AUAGGUGAUGUGGGGCGACGGA				
hsa_circ_0046264 (5′ ... 3′)	CCCGGCUGCCACCACCCUGCCUG	7mer-m8	272	278	84
	|||||||				
hsa-miR-940 (3′ ... 5′)	CCCCUCGCCCCCGGGACGGAA				

Higher context score percentile represents a higher possibility for miRNA to be the target of hsa_circ_0046264

**Table 4:** RNA-binding protein AGO2 sites on hsa_circ_0046264

CircRNA	Tag Name	% Identity	Alignment Length	Mismatches	Gap Openings	Tag Start	Tag End	CircRNA Start	CircRNA End
hsa_circ_0046264	HHRBC_5907_BC3-2of3-SCL_2968_75	100	75	0	0	1	75	373	447
hsa_circ_0046264	HPCB1_18334_G22991.1_79813090_27	100	27	0	0	1	27	397	423
hsa_circ_0046264	HPCB3_10042_G13755.1_79813092_29	100	29	0	0	1	29	397	425

### Hsa_circ_0046264 overexpression down-regulated miR-1245 and up-regulated *BRCA2*

**Fig. 2:**
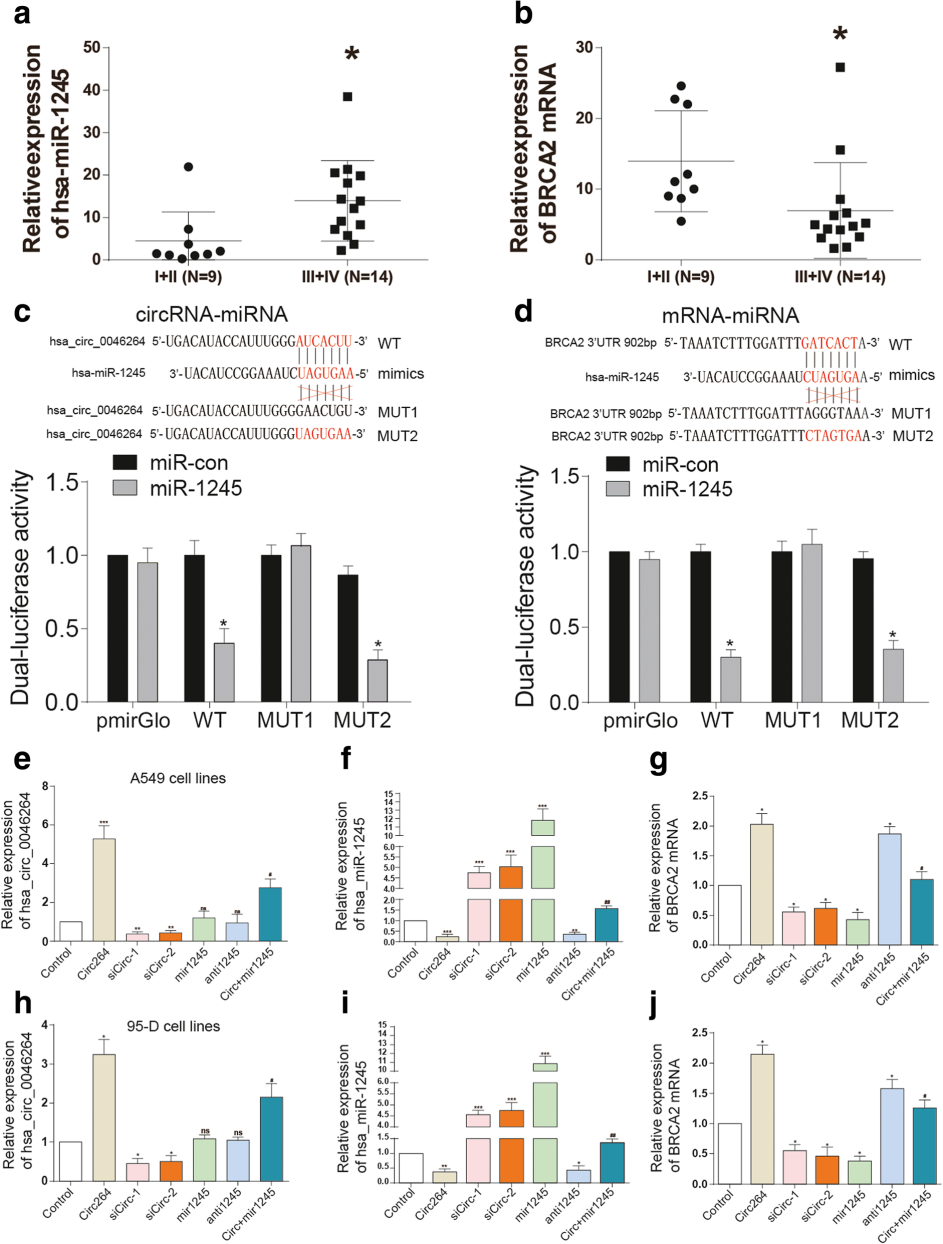
Hsa_circ_0046264 overexpression down-regulated miR-1245 and up-regulated *BRCA2*. **a** The expression level of miR-1245 was higher in advanced lung cancer tissues (stage III and stage IV) than that in early-stage cancer tissues (stage I and stage II). ^*^P < 0.05, compared with I + II (*N* = 9) group with lung cancer tissues in Stage I + II. **b** The expression level of *BRCA2* was lower in early-stage lung cancer tissues than that in advanced cancer tissues. ^*^P < 0.05, compared with I + II (*N* = 9) group with lung cancer tissues in Stage I + II. **c** The dual luciferase activity decreased in HEK293 cells co-transfected with hsa_circ_0046264 WT and miR-1245 mimics, but almost did not change in cells co-transfected with hsa_circ_0046264 MUT and miR-1245 mimics. ^*^P < 0.05, compared with miR-con group. WT: wild type; MUT1: deletion mutation; MUT2: point mutation. **d** The dual luciferase activity decreased in HEK293 cells co-transfected with *BRCA2* 3’UTR WT and miR-1245 mimics, but almost not changed in cells co-transfected with *BRCA2* 3’UTR MUT and miR-1245 mimics. ^*^P < 0.05 compared with miR-con group. (E, H) The relative expression of hsa_circ_0046264 increased in lung cancer cells transfected with the circ-vector pCD2.1-ciR (circ264 group). Control group was transfected with empty pLO-ciR vector. ^***^P < 0.001, compared with control group. ^##^*P* < 0.01, compared with circ264 group. **f**, **i** The relative expression of miR-1245 in lung cancer cells decreased in circ264 group and antimir1245 group but increased in sicirc groups and miR-1245 group. ^***^P < 0.001, compared with control group. ^##^*P* < 0.01, compared with circ264 group. **g**, **j** The expression of *BRCA2* mRNA increased in circ-vector group. ^*^P < 0.05, ^**^P < 0.01, ^***^P < 0.001, compared with control group. ^#^*P* < 0.05, compared with circ264 group

**Fig. 3:**
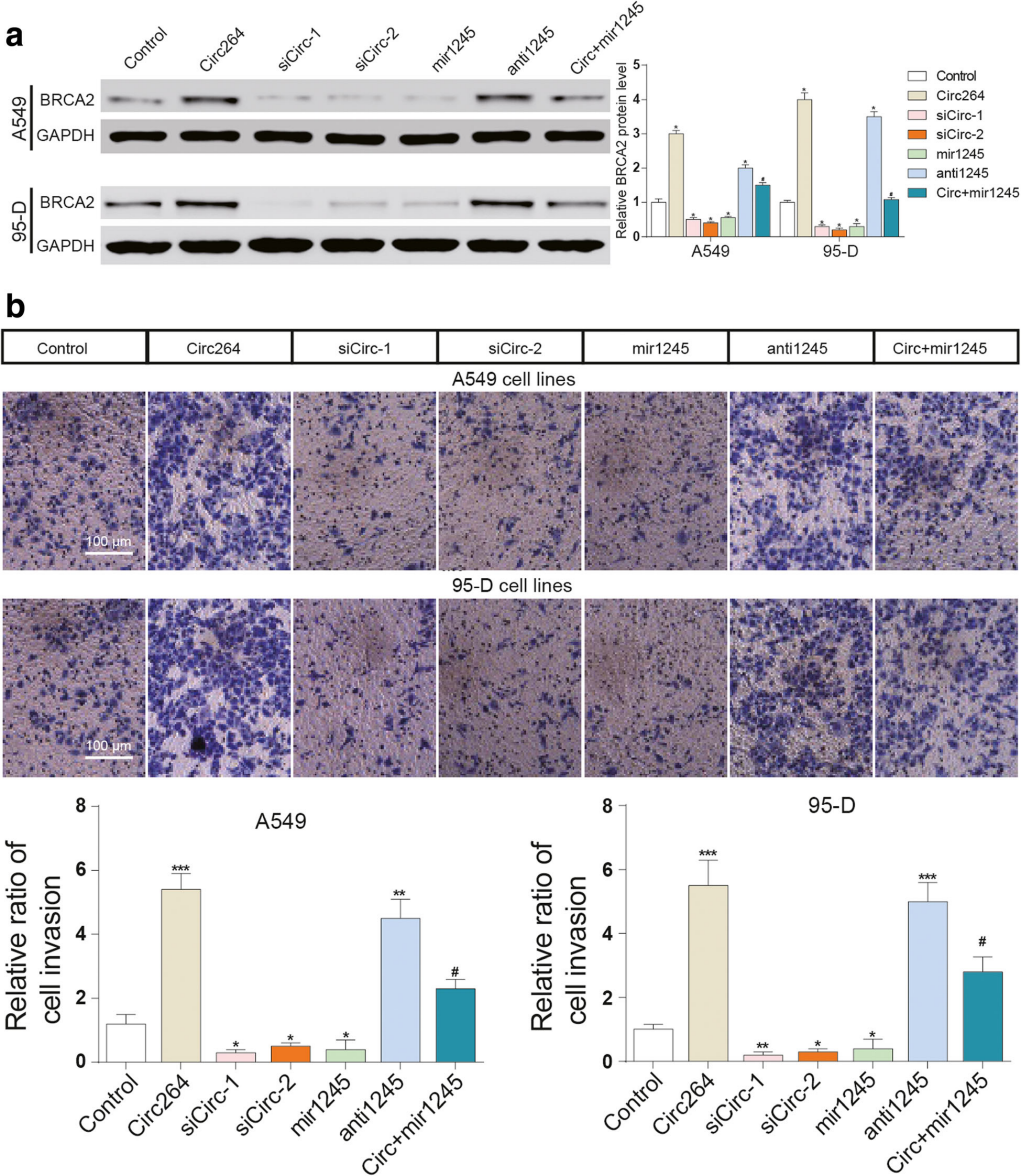
The overexpression of hsa_circ_0046264 promoted BRCA2 expression and cancer cell invasion. **a** The overexpression of hsa_circ_0046264 and the suppression of miR-1245 promoted BRCA2 expression. The simultaneous transfection of hsa_circ_0046264 and miR-1245 did not result in significant change of BRCA2 expression. ^*^P < 0.05, compared with control group. ^#^*P* < 0.05, compared with circ264 group. **b** The overexpression of hsa_circ_0046264 and the suppression of miR-1245 promoted cell invasion. The simultaneous transfection of hsa_circ_0046264 and miR-1245 did not result in significant change of cell invasion. ^*^P < 0.05, ^**^P < 0.01, ^***^P < 0.001, compared with control group. ^#^*P* < 0.05, compared with circ264 group

The expression of miR-1245 was much higher, whereas the expression of BRCA2 mRNA was much lower in III + IV grade cancer tissues than in I + II cancer tissues (Fig. 2a and b). We have verified the target relationship between circ_0046264 and miR-1245 as well as miR-1245 and *BRCA2*. The deletion mutation (MUT1) and point mutation (MUT2) of circ_0046264 and *BRCA2* were used to be as the control of wild type vectors (WT) which showed significant suppressions of luciferase activity by miR-1245(Fig. 2c and d). The transfection efficiency of circ264, sicirc, miR-1245 and antimiR-1245 were tested in both A549 and 95-D cell lines (Fig. 2e and f, h and i). In addition, the influence of modulation of circ_0046264 and miR-1245 expression on BRCA expression was investigated. The results showed that the overexpression of circ_0046264 or the suppression of miR-1245 promoted the expression of BRCA2, whereas the suppression of circ_0046264 led to the suppression of BRCA2 expression (Fig. 2g, j, 3a).

### Hsa_circ_0046264 overexpression suppressed lung cancer development in vitro

**Fig. 4:**
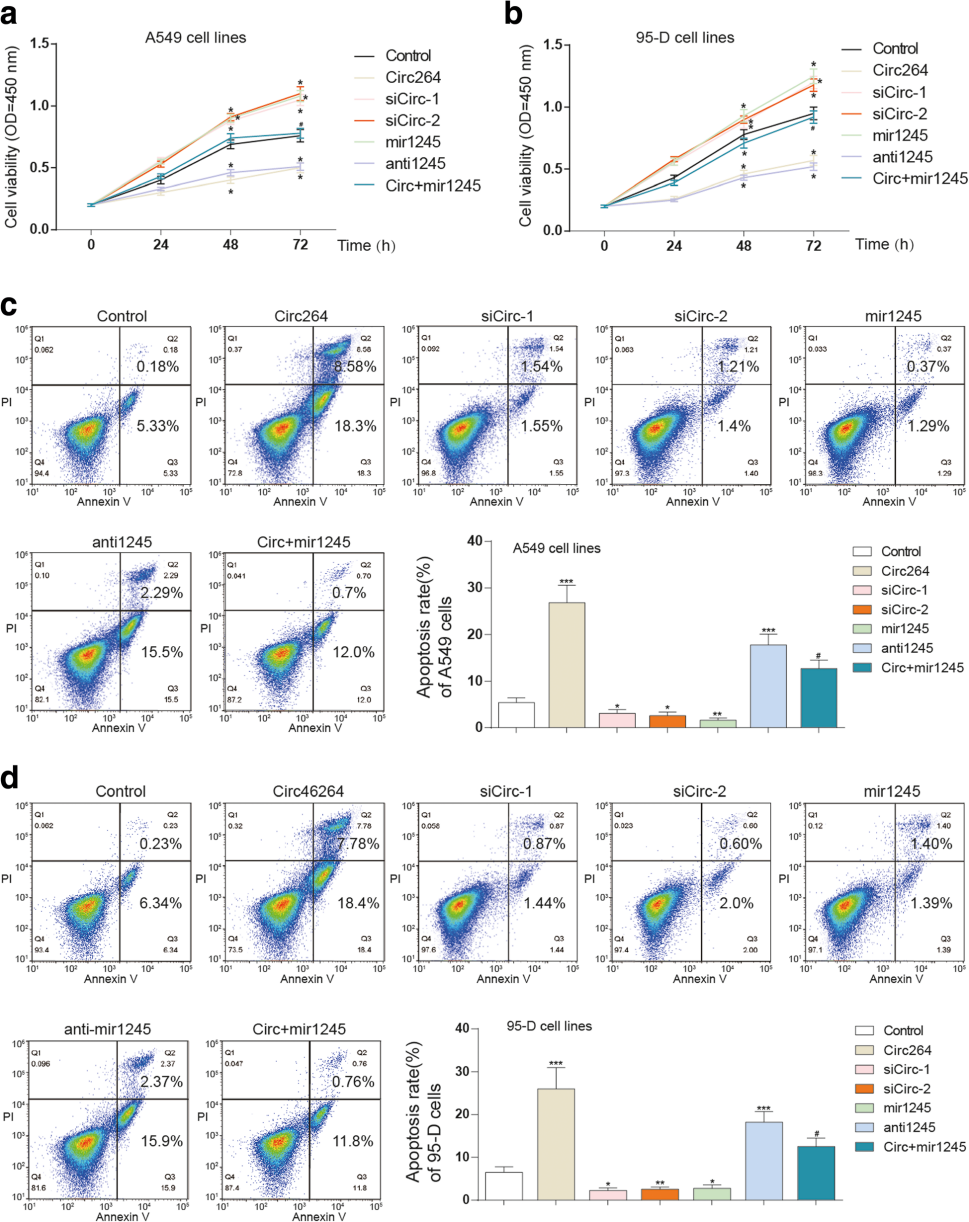
Hsa_circ_0046264 overexpression suppressed lung cancer development in vitro. **a**-**b** Cell viability of A549 and 95-D cells decreased in circ264 group and antimir1245 group but increased in sicirc groups and mir1245 group tested by CCK8 assay. ^*^P < 0.05, compared with control group. **c**-**d** Apoptosis of A549 and 95-D cells was promoted in circ264 group and antimir1245 group but inhibited in sicirc groups and mir1245 group. ^*^P < 0.05, compared with control group

Overexpression of hsa_circ_0046264 or suppression of miR-1245 significantly suppressed the invasion and viability of lung cancer cells; and the suppression of hsa_circ_0046264 or the overexpression of miR-1245 significantly suppressed cell invasion and viability (Fig. 3b, 4a and b). On the contrary, the apoptosis of cancer cells was promoted after being transfected with circ_46264 and inhibited after the cells were knocked down of circ_46264. The overexpression of miR-1245 significantly enhanced cell apoptosis as well (Fig. 4c and d). Besides, γ-H2AX is a marker protein of DNA double-strand damage. The DNA damage of lung cancer cells was also aggravated as more γ-H2AX was observed in circ264 group (Additional file 1: Figure. S1). In brief, the suppressive effect of hsa_circ_0046246 in lung cancer was proved by the in vitro experiments.

### Hsa_circ_0046264 overexpression inhibited tumor growth in vivo

**Fig. 5:**
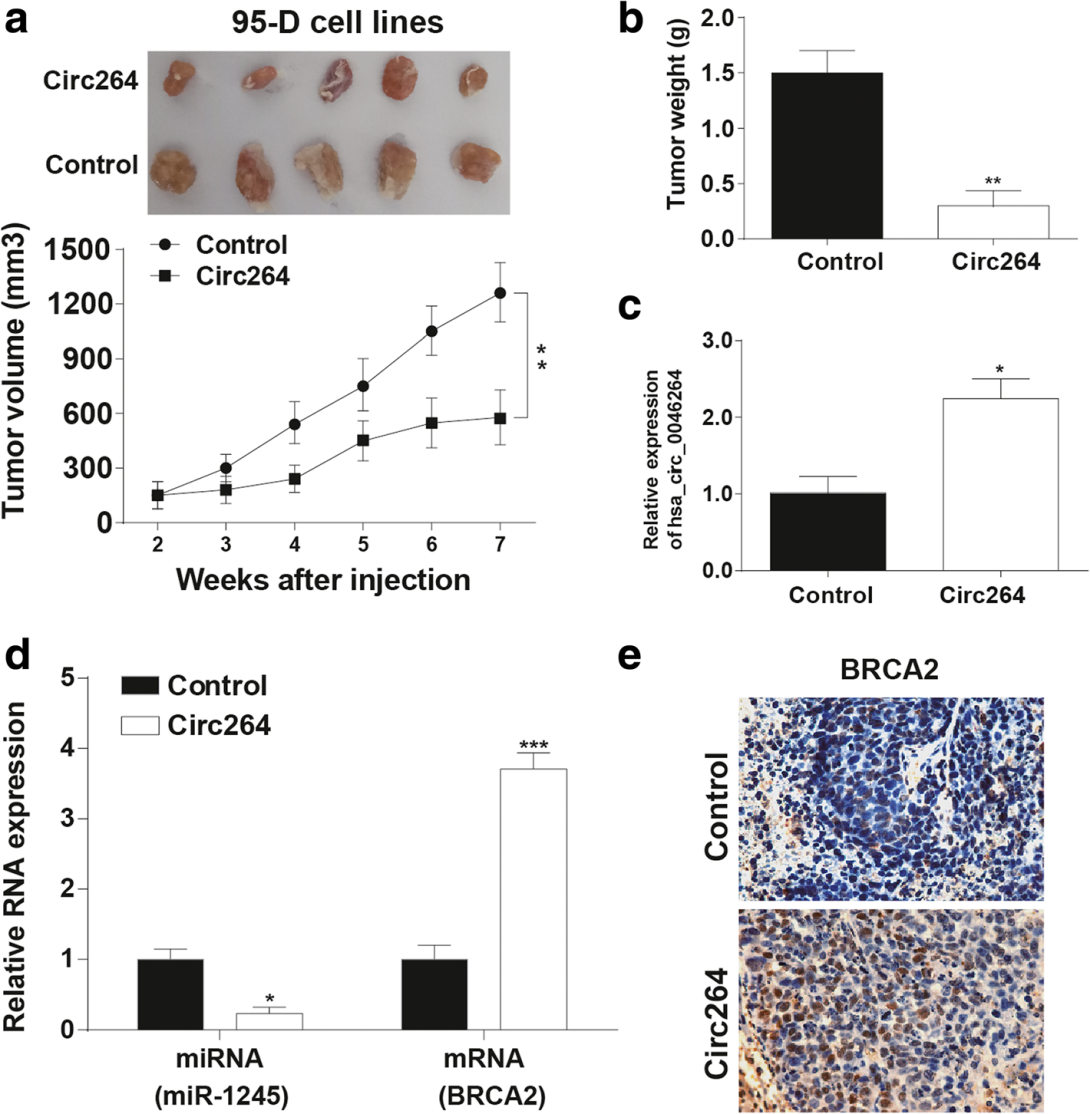
Hsa_circ_0046264 overexpression inhibited tumor growth in vivo. **a**-**b** Tumor growth in hsa_circ_0046264 overexpression group (circ264) was slower than that in control group. Tumor volume and weight in circ264 group were smaller than that in control group. ^**^P < 0.01, compared with control group. **c** The relative expression of hsa_circ_0046264 was higher in circ264 group than that in control group. ^*^P < 0.05, compared with control group. **d** The relative expression of miR-1245 decreased while that of *BRCA2* increased in tumor tissues of circ264 group. ^*^P < 0.05, ^***^P < 0.001 compared with control group. **e** Immunohistochemistry result showed that the expression of *BRCA2* protein increased in tumor tissues of circ264 group

Lung tumors formed in nude mice of hsa_circ_0046264 overexpression group were much smaller than that formed in control group (Fig. 5a). Similarly, hsa_circ_0046264 overexpression also diminished the tumor weight (Fig. 5b). The tumor tissues obtained from the nude mice had an elevated expression level of hsa_circ_0046264 in circ264 group, accompanied by lower miR-1245 and higher *BRCA2* expression levels (Fig. 5c and d). The protein expression level of *BRCA2* also increased, once again proving the up-regulation effect of hsa_circ_0046264 on *BRCA2* expression (Fig. 5e).

## Discussion

Significant down-regulation of hsa_circ_0046264 was found in lung cancer tissues. Overexpression of hsa_circ_0046264 effectively suppressed the proliferation and invasion of lung cancer cells, induced apoptosis. MiR-1245 was suppressed by hsa_circ_0046264, while *BRCA2*, the target gene of miR-1245, was up-regulated. Hsa_circ_0046264 inhibited lung cancer development through acting as a ceRNA which regulated miR-1245 and indirectly activated *BRCA2*.

Hsa_circ_0046264 and *BRCA2* were lowly expressed in lung cancer tissues while miR-1245 was high expressed. Hsa_circ_0046264 could bind miR-1245, and since *BRCA2* was the target gene of miR-1245, *BRCA2* was indirectly up-regulated by hsa_circ_0046264. The regulatory effect of miR-1245on *BRCA2* expression has been reported in previous study as the 3’UTR region of *BRCA2* was combined with miR-1245 [15]. We confirmed the target relationship between miR-1245 and *BRCA2*, and further uncovered their associations with hsa_circ_0046264. Circular RNAs affect cancer processes mainly by serving as ceRNAs, which cross-talk and co-regulate with other genes by competing for binding of miRNAs [20]. CeRNAs involve in the pathogenesis of various common cancers [21]. Based on the altered genes in patients with lung cancer, Jin et al. established a competing endogenous network with several miRNAs and their interacting circRNAs [5]. Here, hsa_circ_0046264 and *BRCA2* shared common target miRNA, miR-1245, and up-regulation of miR-1245 in lung cancer resulted from the low expression of hsa_circ_0046264 caused the down-regulation of *BRCA2*.

The biological functions of hsa_circ_0046264 were inspected in detail in this study. Hsa_circ_0046264 expression was decreased with the deterioration of lung cancer. Overexpression of hsa_circ_0046264 attenuated proliferation, aggravated apoptosis and DNA damage of lung cancer cells as well as inhibited tumor growth. Although circRNAs were suggested to be associated with some kinds of common human cancers, such as circRNAs TCF25 and MYLK in bladder cancer [22, 23], hsa_circ_0005986 and hsa_circ_0004018 in hepatocellular carcinoma [22, 24] and hsa_circ_0000520 in gastric cancer [25], little is known about the roles circRNAs play in lung cancer. Zhao et al. investigated the expression profiles of circRNAs in early-stage lung adenocarcinoma and found that 357 circRNAs were dysregulated and five most significant ones were demonstrated as potential biomarkers [26]. CircRNA ITCH inhibited the activation of Wnt/β-Catenin signaling pathway in lung cancer cells by down-regulating *ITCH*, thus restraining cancer cell proliferation [9]. In contrast, hsa_circ_0013958 and hsa_circ_0000064 were up-regulated in lung cancer, and their aberrant expressions promoted migration and invasion activities of lung cancer cells and attenuated apoptosis [11, 27]. In this study, we identified a novel tumor suppressive circRNA, hsa_circ_0046264. Both in vitro and in vivo results proved its inhibitory effect on the deterioration of lung cancer, suggesting that hsa_circ_0046264 may be a potential target in lung cancer treatment.

Overexpression of hsa_circ_0046264 could diminish the volume and weight of lung tumors formed in nude mice. Meanwhile, miR-1245 expression was reduced in tumor tissues obtained from mice in hsa_circ_0046264 overexpression group, and the mRNA and protein expressions of *BRCA2* were elevated. These results implied that miR-1245 is a potential tumor promotor and *BRCA2* is a suppressor. The influence of miR-1245 in lung cancer has not been identified yet. As for *BRCA2*, it was considered as a susceptibility gene whose mutation was frequently found in breast cancer and ovarian cancer [28, 29]. In lung cancer, Yang et al. suggested that it was associated with a high risk of lung adenocarcinoma [30]. However, according to Lin et al.’s research, polymorphism of rs144848 in *BRCA2* could reduce the lung cancer risk [31]. In this study, we found *BRCA2* was down-regulated in lung cancer tissues and supposed that it was a suppressive gene.

## Conclusion

In summary, hsa_circ_0046264 was proved to be able to inhibit viability and invasion, induce apoptosis of lung cancer cells. It was down-regulated in tumor tissues and successfully restrained tumor growth in vivo. The suppressive effect of hsa_circ_0046264 resulted from its combination with miR-1245, which could indirectly up-regulate miR-1245’s target gene, *BRCA2*. The results demonstrated that hsa_circ_0046264 may be a novel target in lung cancer.

## Additional file

Additional file 1: **Figure S1.** Hsa_circ_0046264 overexpression promoted DNA damage in vitro. (A) The expression of γ-H2AX in circ264 group was significantly higher than in control group in A549 cells. (B) The expression of γ-H2AX in circ264 group was significantly higher than in control group in 95-D cells (TIF 9699 kb).
