# Supplementary figures and images for: Hsa_circ_0046264 up-regulated BRCA2 to suppress lung cancer through targeting hsa-miR-1245
Source: Respir Res. 2018 Jun 11;19:115. doi: 10.1186/s12931-018-0819-7 (PMC5996480; doi:10.1186/s12931-018-0819-7)

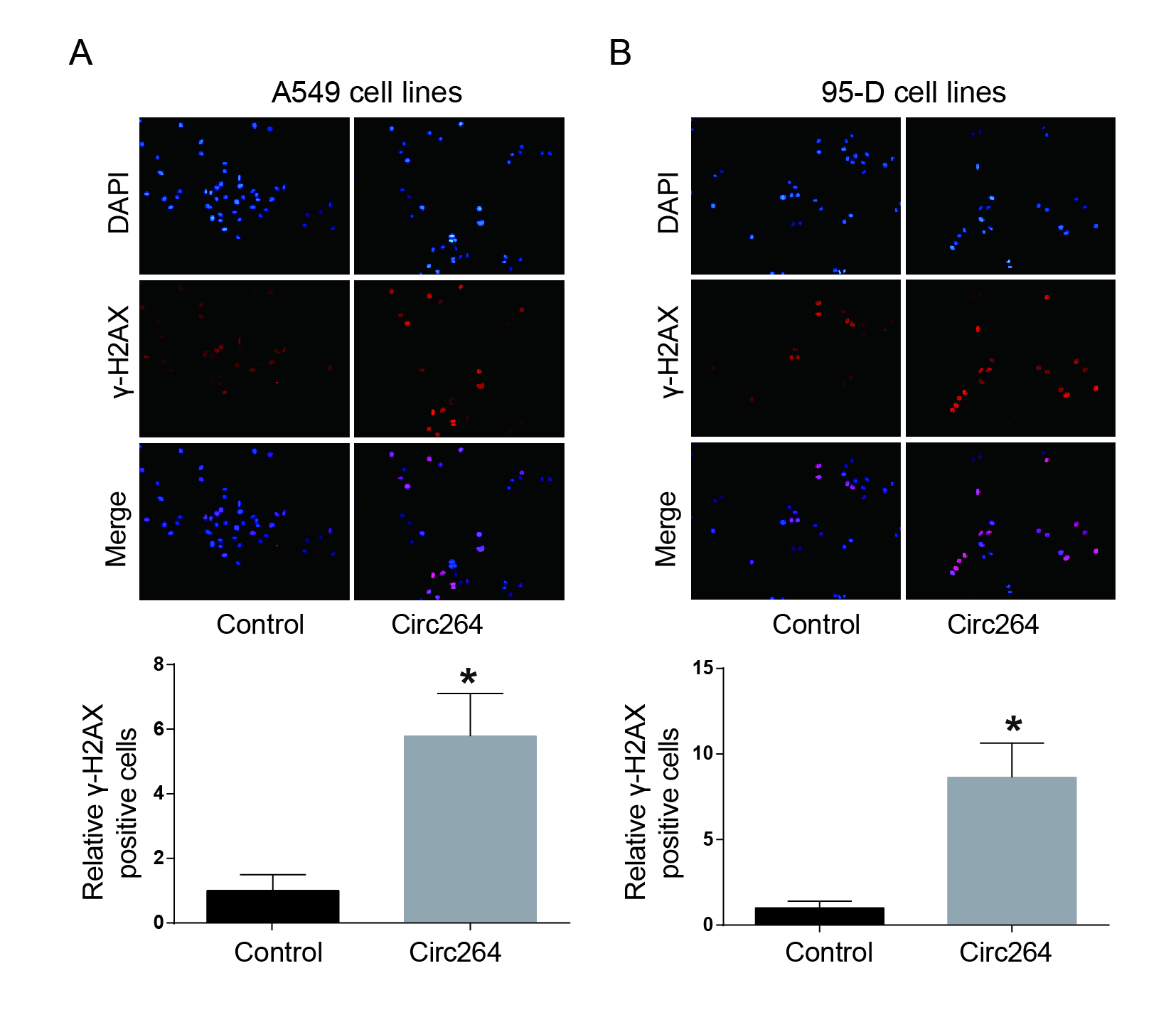

Supplement: Supplementary file 1 — Figure S1. Hsa_circ_0046264 overexpression promoted DNA damage in vitro. (A) The expression of γ-H2AX in circ264 group was significantly higher than in control group in A549 cells. (B) The expression of γ-H2AX in circ264 group was significantly higher than in control group in 95-D cells (TIF 9699 kb). [file 12931_2018_819_MOESM1_ESM.tif]
